# Larvae of *Ixodes ricinus* transmit *Borrelia afzelii* and *B. miyamotoi* to vertebrate hosts

**DOI:** 10.1186/s13071-016-1389-5

**Published:** 2016-02-20

**Authors:** Gilian van Duijvendijk, Claudia Coipan, Alex Wagemakers, Manoj Fonville, Jasmin Ersöz, Anneke Oei, Gábor Földvári, Joppe Hovius, Willem Takken, Hein Sprong

**Affiliations:** Laboratory of Entomology, Wageningen University, PO box 16, 6700 AA Wageningen, The Netherlands; Laboratory for Zoonosis and Environmental Microbiology, National Institute for Public Health and Environment (RIVM), Bilthoven, The Netherlands; Center for Experimental and Molecular Medicine, Academic Medical Center, University of Amsterdam, Amsterdam, The Netherlands; Department of Medical Microbiology, Academic Medical Center, University of Amsterdam, Center for Experimental and Molecular Medicine, Amsterdam, The Netherlands; Department of Parasitology and Zoology, Faculty of Veterinary Science, SzentIstvan University, Budapest, Hungary

**Keywords:** Ixodes ricinus, Larva, Borrelia burgdorferi, Borrelia miyamotoi, Transmission, Infection, Vector, Tick, Rodent

## Abstract

**Background:**

Lyme borreliosis is the most common tick-borne human disease and is caused by *Borrelia burgdorferi sensu lato* (*s.l*.). *Borrelia miyamotoi*, a relapsing fever spirochaete, is transmitted transovarially, whereas this has not been shown for *B. burgdorferi* (*s.l*). Therefore, *B. burgdorferi* (*s.l*) is considered to cycle from nymphs to larvae through vertebrates. Larvae of *Ixodes ricinus* are occasionally *B. burgdorferi* (*s.l*) infected, but their vector competence has never been studied.

**Methods:**

We challenged 20 laboratory mice with field-collected larvae of *I. ricinus*. A subset of these larvae was analysed for infections with *B. burgdorferi* (*s.l*) and *B. miyamotoi*. After three to four challenges, mice were sacrificed and skin and spleen samples were analysed for infection by PCR and culture.

**Results:**

Field-collected larvae were naturally infected with *B. burgdorferi* (*s.l*) (0.62 %) and *B. miyamotoi* (2.0 %). Two mice acquired a *B. afzelii* infection and four mice acquired a *B. miyamotoi* infection during the larval challenges.

**Conclusion:**

We showed that larvae of *I. ricinus* transmit *B. afzelii* and *B. miyamotoi* to rodents and calculated that rodents have a considerable chance of acquiring infections from larvae compared to nymphs. As a result, *B. afzelii* can cycle between larvae through rodents. Our findings further imply that larval bites on humans, which easily go unnoticed, can cause Lyme borreliosis and *Borrelia miyamotoi* disease.

## Background

Lyme borreliosis is the most common vector-borne human disease in the northern hemisphere and is caused by *Borrelia burgdorferi sensu lato* (*s.l*.) [1]. *Borrelia miyamotoi* is an emerging pathogen which can cause relapsing fever, tentatively called *Borrelia miyamotoi* disease in humans [2, 3]. In Europe, both pathogens are transmitted by *Ixodes ricinus*. This tick species hatches from the egg as a larva, which feeds from a vertebrate host before moulting into a nymph. Nymphs feed again from a vertebrate host before moulting into an adult. It is generally believed that larvae of *I. ricinus* are not infected with *B. burgdorferi* (*s.l*.) [4, 5] and can only become infected during a blood meal from an infected host [6] or during a blood meal in the vicinity of an infected nymph feeding on an uninfected host, known as co-feeding [7, 8]. The infected engorged larvae then moult into infected nymphs, which can transmit the spirochaetes to new hosts [9]. Rodents are the most frequently used hosts by larvae and are reservoirs for *B. afzelii* [10]. However, *B. burgdorferi* (*s.l*.) is also, but rarely, found in questing larvae [11–14]. *Borrelia miyamotoi,* on the other hand, can be transmitted transovarially from female tick to larva [15] but is only shortly maintained in rodents [16]. Presence of a pathogenic microorganism in a tick does not necessarily mean that the tick is also competent as a vector [17]. On rodents, larval tick burden is much higher than nymphal tick burden, while adult *I. ricinus* rarely feed on rodents [18]. Humans are also bitten by larvae, with an estimated of at least 30,000 larval bites - out of 1.1 million tick bites - in The Netherlands annually [19]. Therefore, even a low *B. burgdorferi* (*s.l*) infection rate in larvae will be of importance to the enzootic transmission cycle of *B. burgdorferi* (*s.l*) [14] and human disease risk.

The aim of this study was to examine whether the larvae of *I. ricinus* can transmit *B. burgdorferi* (*s.l*) and *B. miyamotoi* to vertebrate hosts.

## Methods

### Study organisms

Twenty-eight male Naval Medical Research Institute (NMRI) mice 2 months-old were used. Experiment 1 consisted of 10 treatment and two control mice and experiment 2 consisted of 10 treatment and six control mice. Mice were housed individually in unmodified Makrolon type II cages with *ad libitum* water and food. Cages were suspended over pans filled with water and with petroleum jelly on the edge.

Larvae of *I. ricinus* were captured at five locations (Buunderkamp, Grebbeberg, Sysselt, Bilderberg and Planken Wambuis) near Wageningen, The Netherlands (Table 1). All larvae were captured in June-September 2014 within 2 weeks prior to each challenge (see below) or pathogen detection. All locations consisted of mixed forests dominated by Scots pine (*Pinus sylvestris*). A 1x1m blanket was dragged over the vegetation [20] and attached larvae were collected using an aspirator. A subset of these questing larvae were individually analysed for natural infections with *B. burgdorferi* (*s.l*) and *B. miyamotoi*. Larvae were stored in groups of 200 in ventilated 50 ml tubes and stored at room temperature, 90 % relative humidity (RH) and natural day length until further use. Tick life-cycle stage was confirmed using a microscope.

**Table 1:** Origin, mouse numbers and infection rates of questing larvae

Location	Coordinates	Mouse	*B. burgdorferi*	*B. miyamotoi*
Buunderkamp	52°00'46"N, 5°45'42"E	1–2, 13–14	1.12 (3/267)	2.25 (6/267)
Grebbeberg	51°57'10"N, 5°35'25"E	3–4, 15–16	1.29 (3/233)	0.43 (1/233)
Sysselt	52°01'40"N, 5°41'26"E	5–6, 17–18	0.39 (1/257)	0.39 (1/257)
Bilderberg	51°59'55"N, 5°48'34"E	7–8, 19–20	0 (0/362)	2.49 (9/362)
Planken Wambuis	52°01'36"N, 5°48'55"E	9–10, 21–22	0.59 (2/337)	3.56 (12/337)
Mean			0.62 (9/1456)	1.99 (29/1456)

Questing larvae were collected at five locations. Coordinates, mouse numbers and *B. burgdorferi* (*s.l*) and *B. miyamotoi* infection rates (infected/analysed) of questing larvae are shown per location

### Challenges with larvae

Mice were challenged with 200 larvae on the head and neck under anaesthesia (60 mg/kg pentobarbital, i.p.) at 2-week intervals. In experiment 1, five groups of two mice were subjected to four challenges with 200 larvae collected from five different locations (Table 1). Two control mice received no larval challenge. In experiment 2, five groups of two mice were similarly subjected to three challenges with 200 field-collected larvae while six control mice were not challenged. Engorged larvae were collected from the pans with water four and seven days after each challenge and dried on filter paper for 2 h. These larvae were then housed individually in ventilated 0.2 ml Eppendorf tubes at 20 °C, 90 % RH and a day length of 14 h. Engorged larvae of experiment 1 were checked monthly, and 2 months after moulting, the emerged nymphs were stored at −20 °C until further use. Larvae that did not moult and larvae from experiment 2 were excluded from the molecular analysis.

### Collection of mouse tissue

An ear biopsy was collected from each mouse with sterile scissors and tweezers 1 week before challenge 1. One week after challenge 4 in experiment 1 and 3 weeks after challenge 3 in experiment 2, mice were sacrificed by cardiac bleed followed by cervical dislocation under anaesthesia (60 mg/kg pentobarbital, i.p.), after which two ear biopsies and a spleen sample were collected. All tissue samples were stored in 70 % ethanol at −20 °C until further use.

### Ethical approval

All experiments were approved by the Ethical committee of Wageningen University (number 2013136).

### Natural tick burden on field-collected rodents

In 2013 and 2014, rodent life traps were placed with 5 m inter-trap distance in grids of 12*12 and 6*12, respectively, at two locations in a forest near Wageningen, The Netherlands (51°59'35.43"N, 5°43'42.06"E) and (51°59'37.22"N, 5°43'22.08"E). Traps were set at 15:30 h and inspected the next day at 08.30 h at 3-week intervals from May till November. Tick burdens of trapped wood mice (*Apodemus sylvaticus*) and bank voles (*Myodes glareolus*) were determined by searching the head, ears, snout, belly, legs, armpits, throat and tail.

### Pathogen detection

In experiment 2, mouse spleens and ears collected at the end of the experiment were cultured in MKP-F medium as described in detail previously [21]. In short: a 4 mm tissue biopsy was disinfected and placed in 7 ml Modified Kelly-Pettenkofer medium containing rifampicin, fosfomycin and amphotericin B as antibiotics. Cultures were incubated at 33 °C and checked weekly for motile spirochaetes using a dark-field microscope at 250x magnification. After 3 weeks 500 μl of medium was inoculated into a new tube containing 7 ml of culturing medium. This was repeated three times. DNA from the cultures containing motile spirochaetes, tissue samples and moulted nymphs were extracted using the Qiagen DNeasy Blood & Tissue Kit [22]. DNA from questing larvae was extracted by alkaline lysis [23]. The presence of *B. burgdorferi* (*s.l*) DNA was detected with a duplex quantitative PCR using fragments of the outer membrane protein A gene and the flagellin B gene as targets [24]. In the same qPCR, *B. miyamotoi* could specifically be detected with primers and probe based on the flagellin gene for detection of the bacteria. The presence of *Neoehrlichia mikurensis* and *Anaplasma phagocytophilum* was detected as described [22]. Multi-Locus Sequence Typing on the *Borrelia* cultures was performed as described [25].

### Data analysis

Infection rates of questing larvae and emerged nymphs were compared using a generalized linear model (GLM, assuming binomial distribution with logit link function) in SAS statistical software, version 9.3.

## Results

### Infection rates of field-collected larvae in nature and after feeding on laboratory mice

A subset of 1456 field-collected questing larvae were analysed for infection. In experiment 1, 1897 engorged field-collected larvae that fed on laboratory mice were collected, from which 1823 (96 %) moulted into nymphs. *Borrelia burgdorferi* (*s.l*) infection rate increased from 0.62 % (9/1,456) in questing larvae to 1.65 % (30/1,823) in larvae that moulted into nymphs after feeding (Tables 1 and 2, likelihood ratio chi-square 7.79, df = 1, *P* = 0.005). *Borrelia miyamotoi* infection rate of questing larvae (1.99 %, 29/1,456) and larvae that moulted into nymphs after feeding (1.76 %, 32/1,823) did not differ (likelihood ratio chi-square 0.25, df = 1, *P* = 0.619). No questing larvae were co-infected with *B. burgdorferi* (*s.l*) and *B. miyamotoi*. Two nymphs (mouse 9, challenge 2 and mouse 10, challenge 4) were co-infected with *B. burgdorferi* (*s.l*) and *B. miyamotoi*. In experiment 2, 2308 engorged field-collected larvae were collected, which were not further analysed.

**Table 2:** Infection rates (infected/analysed) of ticks after feeding on mice of experiment 1

	Mouse	Challenge 1	Challenge 2	Challenge 3	Challenge 4	PCR-positive tissues
*B. burgdorferi* (*s.l*)	1	1.3 (1/75)	0 (0/81)	2.2 (1/45)	0 (0/48)	-
	2	0 (0/32)	0 (0/39)	3.8 (1/26)	0 (0/13)	-
	3	1.7 (1/60)	0 (0/64)	0 (0/34)	0 (0/34)	-
	4	0 (0/47)	0 (0/31)	0 (0/32)	0 (0/26)	-
	5	0 (0/80)	0 (0/47)	0 (0/55)	1.3 (1/75)	-
	6	0 (0/44)	0 (0/58)	0 (0/37)	0 (0/20)	-
	7	0 (0/84)	0 (0/39)	0 (0/36)	0 (0/44)	-
	8	2.2 (1/45)	2.1 (1/47)	0 (0/51)	0 (0/4)	-
	9	0 (0/73)	2.9 (1/35)	0 (0/15)	0 (0/21)	-
	10	0 (0/102)	3.2 (2/63)	0 (0/33)	71.4 (20/28)	Ear
	total	(3/642)	(4/504)	(2/364)	(21/313)	
*B. miyamotoi*	1	0 (0/75)	2.5 (2/81)	0 (0/45)	2.1 (1/48)	-
	2	0 (0/32)	7.7 (3/39)	0 (0/26)	0 (0/13)	Spleen
	3	3.3 (2/60)	0 (0/64)	0 (0/34)	0 (0/34)	Spleen
	4	0 (0/47)	0 (0/31)	0 (0/32)	3.8 (1/26)	-
	5	1.3 (1/80)	0 (0/47)	0 (0/55)	1.3 (1/75)	-
	6	0 (0/44)	0 (0/58)	2.7 (1/37)	0 (0/20)	-
	7	0 (0/84)	0 (0/39)	0 (0/36)	0 (0/44)	-
	8	0 (0/45)	0 (0/47)	0 (0/51)	0 (0/4)	-
	9	17.8 (13/73)	14.3 (5/35)	0 (0/15)	0 (0/21)	-
	10	0 (0/102)	0 (0/63)	3 (1/33)	3.6 (1/28)	-
	total	(16/642)	(10/504)	(2/364)	(4/313)	-

Last column shows PCR-positive tissues after challenge 4

### Pathogen transmission by field-collected larvae

All mice were not infected with *B. burgdorferi* (*s.l*) and *B. miyamotoi* at the start of the experiments and all control mice were uninfected at the end of the experiments (Table 3). In experiment 1, 1 week after four challenges with field-collected larvae, 1 out of 10 mice was positive for *B. afzelii* and 2 out of 10 mice for *B. miyamotoi*. In experiment 2, 3 weeks after three challenges with field-collected larvae, 1 out of 10 mice was positive for both *B. afzelii* and *B. miyamotoi*. We were able to isolate and culture live spirochaetes for more than three passages from the ears of this infected mouse. The motile spirochaetes in this culture and the *B. burgdorferi* (*s.l*)-positive tissue samples and nymphs were all identified as *B. afzelii* by molecular typing. In addition, in experiment 2, 1 out of 10 mice was positive for *B. miyamotoi*. None of the rodent samples were infected with *Anaplasma phagocytophilum* or *Neoehrlichia mikurensis* (data not shown).

**Table 3:** Infections in mice of experiments 1 and 2

	Mouse	Treatment	Before challenges	After challenges
Experiment 1	1	T	-	-
	2	T	-	Bm (S)
	3	T	-	Bm (S)
	4	T	-	-
	5	T	-	-
	6	T	-	-
	7	T	-	-
	8	T	-	-
	9	T	-	-
	10	T	-	Ba (E)
	11	C	n.a.	-
	12	C	n.a.	-
Experiment 2	13	T	-	-
	14	T	-	-
	15	T	-	-
	16	T	-	Ba (E), Bm (S, E)
	17	T	-	-
	18	T	-	-
	19	T	-	-
	20	T	-	-
	21	T	-	-
	22	T	-	Bm (S)
	23	C	-	-
	24	C	-	-
	25	C	n.a.	-
	26	C	n.a.	-
	27	C	n.a.	-
	28	C	n.a.	-

*T* treatment mouse, challenged with larvae, *C* control mouse, *n.a.* not analysed, *S* Spleen, *E* Ear biopsy, *Ba Borrelia afzelii, Bm Borrelia miyamotoi*

- = uninfected

### Natural tick burden on field-collected rodents

In total, 335 wood mice and 521 bank voles were trapped. Total number of nymphs on these rodents was 60 (mean 0.07 ± 0.013) and total number of larvae 3157 (mean 3.69 ± 0.235), resulting in a nymph to larva ratio of 1:52.6.

## Discussion

We showed that larvae of *I. ricinus* can transmit Lyme disease spirochaetes. An abstract in a conference book from 2002 claimed this finding before, but it was never published in a full research article [26]. The possibility to culture *B. afzelii* from several organs of the rodents shows that the spirochaetes were viable and infectious. The rodents infected with *B. afzelii* subsequently facilitated transmission to other larvae, which successfully moulted to infected nymphs (71.4 % infectivity during the last challenge). This resulted in a higher infection rate in the emerged nymphs compared to the infection rate of questing larvae. These results are in contradiction to the general idea that *B. burgdorferi* (*s.l*) can only be transmitted by nymphs (and adults), which acquired the infection as a larvae (or nymphs) during feeding [6–8]. We have shown that 0.62 % of the larvae in nature is infected with *B. burgdorferi* (*s.l*), whereas this is about 10 % (range 0–60 %) for nymphs [27, 28]. On rodents, which are the main maintenance host for larvae and reservoir host of *B. afzelii,* we found a nymph to larva ratio of 1:52.6, which is in accordance with the ratios found by Gassner *et al.* [18] and Pisanu *et al.* [29]. This means that among 1000 feeding nymphs there are 100 infected (10 %) and at the same time 52,600 larvae feed on the same rodent population from which 326 (0.62 %) are infected. As a result, assuming comparable transmission efficiencies for larvae and nymphs, larval contribution to rodent infections is approximately three times higher than the nymphal contribution. When transmission efficiency from *I. ricinus* to rodents is considered to be 100 % for nymphs [30] and 10 % for larvae (1 out of 10 larvae, Table 2), larvae are still responsible for a quarter of the infections in rodents. The transmission efficiencies of larvae and nymphs need further research. Regardless of these transmission efficiencies, our results show that *B. afzelii* can also cycle between larvae through rodents without the interference of nymphs. Infection rates of *B. burgdorferi* (*s.l*) in questing larvae can even be up to 25.8 %, as found in Germany [14], increasing the importance of larval infection.

**Fig. 1:**
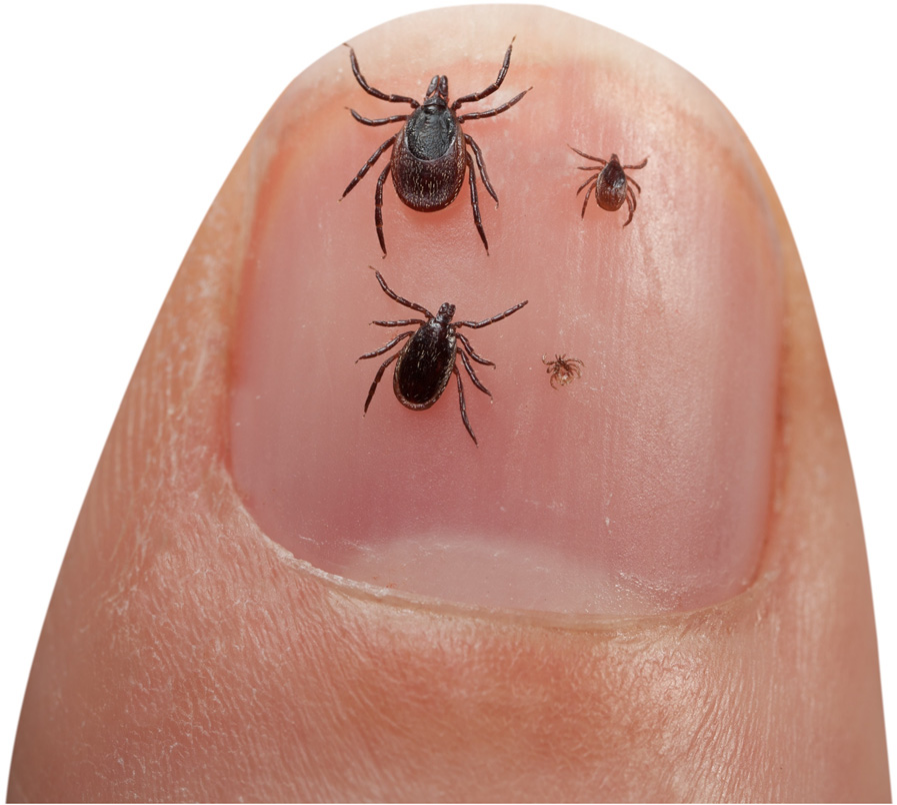
Developmental stages of *I. ricinus*. From left to right and top to bottom: Adult female, nymph, adult male, larva (Picture: Hans Smid)

In The Netherlands, 1.1 million people are bitten by one or more ticks annually [19]. Larvae are responsible for 1.3 to 4.2 % of human tick bites [31–33], and it is estimated that in The Netherlands 30,000 people were bitten by larvae. A significant number of Lyme borreliosis patients did not recognize or remember being bitten by a tick, which might especially be the case for larval ticks due to their minute size (Fig. 1). Our findings provide a reasonable explanation when Lyme borreliosis patients have not noticed a tick bite.

As far as we know, we also showed for the first time that larvae of *I. ricinus* can transmit *B. miyamotoi*. We found a *B. miyamotoi* infection rate of 2 % in larvae, which is comparable to the infection rate found in nymphs [32, 34]. This suggests a potentially higher contribution of larval tick bites to tick-borne relapsing fever in humans compared to larval contribution to Lyme borreliosis. Other studies have previously suggested an important role for larvae in the transmission of *B. miyamotoi* based on infections coinciding with larval activity and infestation [35, 36]. The contribution of larval transmission to rodents and from rodents to larvae to the enzootic life cycle of *B. miyamotoi* appears to be low. We did not find an increase in the infection rate of larvae with successive challenges, even in the two mice (2 and 3) that were *B. miyamotoi* positive after challenge 4. Infection rate of the emerged nymphs was not higher compared to the infection rate in questing larvae. In addition, mouse 9 was infectious to feeding larvae during challenge 1 and challenge 2, but was PCR negative after challenge 4, suggesting that, in contrast to *B. afzelii*, *B. miyamotoi* does not cause a persistent infection in rodents [16].

In experiment 1, 7 out of 10 mice were exposed to larvae that were (after feeding) *B. afzelii*-positive and 9 out of 10 mice to *B. miyamotoi*-positive larvae. However, only 1 out of 10 and 2 out of 10 mice were positive after challenge 4 for *B. afzelii* and *B miyamotoi*, respectively. For *B. afzelii*, this can indicate a low transmission efficiency from larva to rodent, whereas the *B. miyamotoi* infections could have been cleared from the rodents before the dissections after challenge 4. In addition, mice were only naïve during the first challenge because they were challenged 4 times with larvae. This could have resulted in the production of antibodies against *B. miyamotoi* making the mice resistant to infections during challenge 4.

Knowing now that larvae of *I. ricinus* can transmit *B. afzelii* to rodents, the next question is how larvae can acquire the infection. In theory, larvae may acquire spirochaetes through transovarial transmission from female tick to larvae or through a partial blood meal from a rodent, after which the larva quests for a second host. Transovarial transmission, however, has never been demonstrated for *B. burgdorferi* (*s.l*) but is indeed considered an important mechanism for *B. miyamotoi* [4, 5, 15]. Partial feeding on an infected rodent for only eight hours incidentally resulted in *B. burgdorferi* (*s.l*) infection of *I. scapularis* larvae [37]. In addition, nymphs that have fed for up to 48 h on one host can thereafter infect another host without first moulting into an adult [38]. Partially engorged larvae, however, were unable to transmit *B. burgdorferi* (*s.l*) to a host during a subsequent feeding, which was argued to be because of a too low spirochete load for efficient transmission [37]. However, an inoculum of only 10 spirochaetes has been demonstrated to be sufficient to successfully infect a rodent [39]. Partially-engorged larvae can arise when feeding larvae are detached due to grooming or due to an immune response or death of the host [40–42]. Larvae appear to be unfed macroscopically after up to 18 h of feeding, during which they may have acquired an infection [37]. Partially-engorged questing nymphs are occasionally observed in the field (GD, pers. obs.). However, frequency of naturally occurring partially engorged and *B. burgdorferi* (*s.l*)-infected larvae is unknown. Both mechanisms (transovarial transmission and partial feeding) seem equally unlikely. The route via which larvae can acquire a *B. burgdorferi* (*s.l*) infection needs further research.

## Conclusions

Larvae of *I. ricinus* can transmit *B. afzelii* and *B. miyamotoi*. This finding changes the current view on the enzootic lifecycle of *B. afzelii* and the current public health dogma that larval bites cannot cause Lyme disease in humans.
